# 3D FEM comparison of lingual and labial orthodontics in *en masse* retraction

**DOI:** 10.1186/s40510-014-0038-9

**Published:** 2014-05-30

**Authors:** Luca Lombardo, Giuseppe Scuzzo, Angela Arreghini, Özge Gorgun, Yıldız Öztürk Ortan, Giuseppe Siciliani

**Affiliations:** Department of Orthodontics, University of Ferrara, Via Montebello 31, Ferrara, 44100 Italy; Incirli Caddesi, Hur Sokak 1/5, Bakirkoy, Istanbul; Department of Orthodontics, Istanbul University, Istanbul, Turkey

**Keywords:** Finite element modeling, Lingual brackets, En masse retraction

## Abstract

**Background:**

The aim of this study was to compare displacements and stress after *en masse* retraction of mandibular dentition with lingual and labial orthodontics using three-dimensional (3D) finite element models (FEM).

**Methods:**

A 3D FEM of each lower tooth was constructed and located as appropriate to Roth's prescription. The 0.018-in. GAC Roth Ovation labial and Ormco 7th Generation lingual brackets were virtually bonded to the lower teeth and threaded with 0.018 × 0.025- and 0.016 × 0.022-in. SS labial (Tru-Arch form, small size) and lingual (mushroom) archwires. *En masse* retraction was simulated by applying 300 g of distal force from the canine to the second premolar on the 0.016 × 0.022-in. SS labial and lingual archwires. The type of finite element used in the analysis was an eight-noded brick element. The Algor program (Algor Inc., Pittsburgh, PA, USA) was used to calculate the strains and displacements at each nodal point.

**Results:**

Lingual tipping and extrusion of the anterior dentition occurred with both archwires. At the premolars and first molars, intrusion, lingual movements, and lingual tipping were seen with the labial archwire, while intrusion was accompanied by labial movements, mesial tipping, and buccal rotation with lingual mechanics.

**Conclusions:**

Lingual vs. labial bracket placement influences the pattern of tooth movement, but the stress that occurs around the teeth can be accurately mapped using a 3D FEM model.

**Electronic supplementary material:**

The online version of this article (doi:10.1186/s40510-014-0038-9) contains supplementary material, which is available to authorized users.

## Background

Lingual appliances marked a great leap forward in aesthetic orthodontics, thanks to their unobtrusiveness, and recent improvements in terms of indirect lingual bracket bonding, new archwire materials, and computerized planning systems have made the technique even simpler and more precise [1]. Nevertheless, lingual appliances have their own peculiar biomechanics, distinct from that of conventional orthodontics, and special care must be taken in their application. In particular, for aesthetic reasons, the six anterior teeth are generally retracted as a unit in the lingual technique, so as not to create any space between canines and lateral incisors. This clinical procedure appears to offer better anchorage on the lower posterior teeth than labial treatment, due to the different point of force application. That being said, mesial movement of the posterior teeth is known to be more problematic in lingual orthodontics than in the labial technique [2], as the periodontal stresses generated by orthodontic forces are transferred to the alveolar bone, leading to resorption in compressed regions and apposition where the bone is under tensile stress.

It is very difficult to measure clinically the stress induced at various locations within the root by different types of orthodontic tooth movement. Although a variety of traditional analytical and experimental methods for analyzing dental stresses, such as photoelasticity, interferometric holography, and strain gauges, have shed some light on the mechanism of orthodontic tooth movement, they have been unable to clarify the micro-environmental changes around the periodontal ligament (PDL) and within the bone [3]. However, the finite element method (FEM) described by Zienkiewicz has been used to investigate a wide range of dentistry topics, including tooth structure [4, 5], biomaterials and restorations [5–8], and dental implants and root canals [4, 5], and may elucidate the reaction of the teeth, periodontal ligament, alveolar bone, etc. to orthodontic loading. FEM is a mathematical method in which the shape of complex geometric objects and their physical properties are computer-constructed. Physical interactions of the various components of the model can then be calculated in terms of stress and strain, a detailed information which is difficult to obtain by any other experimental or analytical means due to the interaction of anatomical structures with the surrounding tissue [7, 9]. In order to capitalize on this powerful computational tool, we set out to make three-dimensional (3D) FEM models of the lower jaw and dentition, in order to map and compare the initial displacements and stress produced by simulated *en masse* retraction performed with lingual and labial appliances.

## Methods

A 3D FEM of each lower tooth was constructed manually according to the detailed dimensions and morphology supplied by Wheeler's Dental Atlas [10]*.* Roth prescription was used to establish the angulations and inclinations of each tooth with reference to Andrews' facial axis (FA) point [11]. Virtual models of 0.018-in. lower GAC Roth Ovation labial (DENTSPLY GAC International, Islandia, NY, USA) and Ormco 7th Generation lingual (ORMCO CORPORATIONE, Orange, CA, USA) brackets and 0.018 × 0.025- and 0.016 × 0.022-in. SS labial (Tru-Arch form, small size, Ormco) and lingual (mushroom) archwires were constructed, and labial brackets and tubes were placed on the teeth in their ideal positions. The ideal lower dentition was established by inserting a .018 × 0.025-in. SS labial full-dimension archwire in the slots of the labial brackets and tubes. When ideal dentition was achieved, a 0.018 × 0.025-in. SS mushroom arch wire was used for placing lingual brackets and tubes in their proper positions.

The material properties of all dentoalveolar structures were assumed to be isotropic and homogeneous. The PDL was considered to have a uniform thickness of 0.25 mm around the root, and the thickness of the alveolar cortical bone was taken to be 1.0 mm. Young's moduli and Poisson's ratios for the materials were assumed as the average values reported in the literature, as shown in Table 1[12]. Despite the fact that analytical deformations may increase significantly over time, for simplification purposes, the force and deformation characteristics were assumed to be time independent.

**Table 1:** **Properties of simulated materials**

	Young's modulus (N/mm)	Poisson's ratio
Cancellous bone	1370	0.30
Cortical bone	13700	0.26
PDL	0.6668	0.49
Tooth	20000	0.30
Bracket and SS wire	214000	0.30

**Figure 1:**
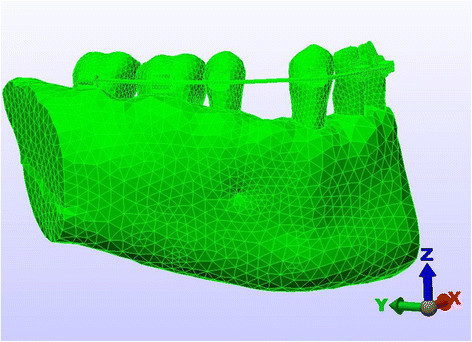
**Artificial boundary of the FEM (labial technique).**

**Figure 2:**
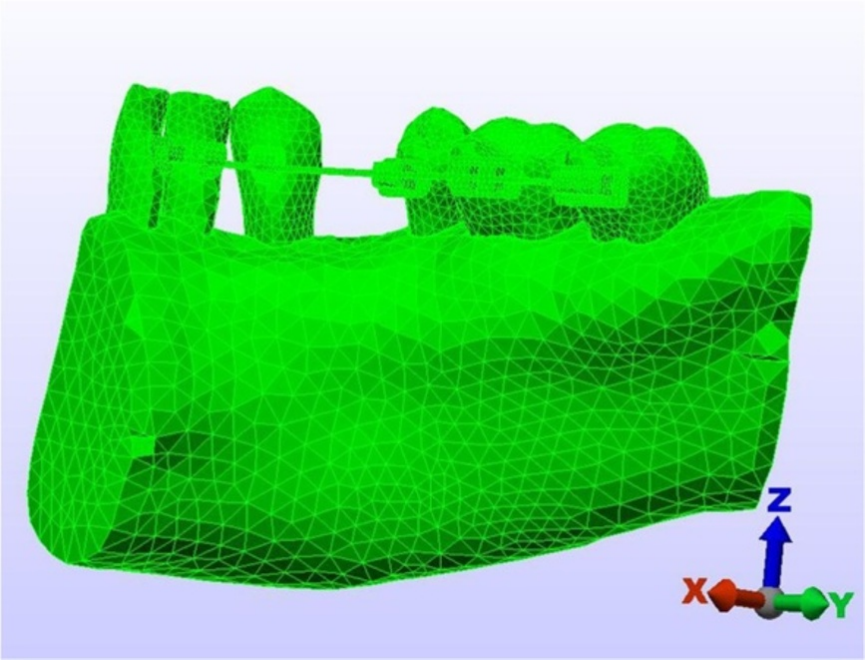
**Artificial boundary of the FEM (lingual technique).**

The type of finite element used in the analysis was an eight-noded brick element, and the mathematical model comprised a total of 128,298 elements (38,606 nodes). Although the cancellous bone extends quite a distance within the alveolar bone, the finite element model generated was restricted to a certain zone beyond the cortical bone. The cancellous bone has been truncated horizontally approximately a few millimeters (1/3 of the tooth length) below the tooth roots, making an artificial lower boundary (Figures 1 and 2). All nodes at this artificial boundary are assumed to be constrained in the manner of a movable hinge.

After the model was completed, boundary conditions were defined at all peripheral nodes of the bone, giving them 0° of movement in all directions. Link elements were defined between the nodes on the mesial and distal ends of the bracket to simulate the bracket ligation and prevent the archwire coming out of the slots. To simulate the friction force, contact elements were defined between contact surfaces of the archwire and bracket slots, assuming a friction coefficient of 0.2. During *en masse* retraction, the anterior and posterior teeth acted as a unit, the segments being linked via eight virtual ligations.

**Figure 3:**
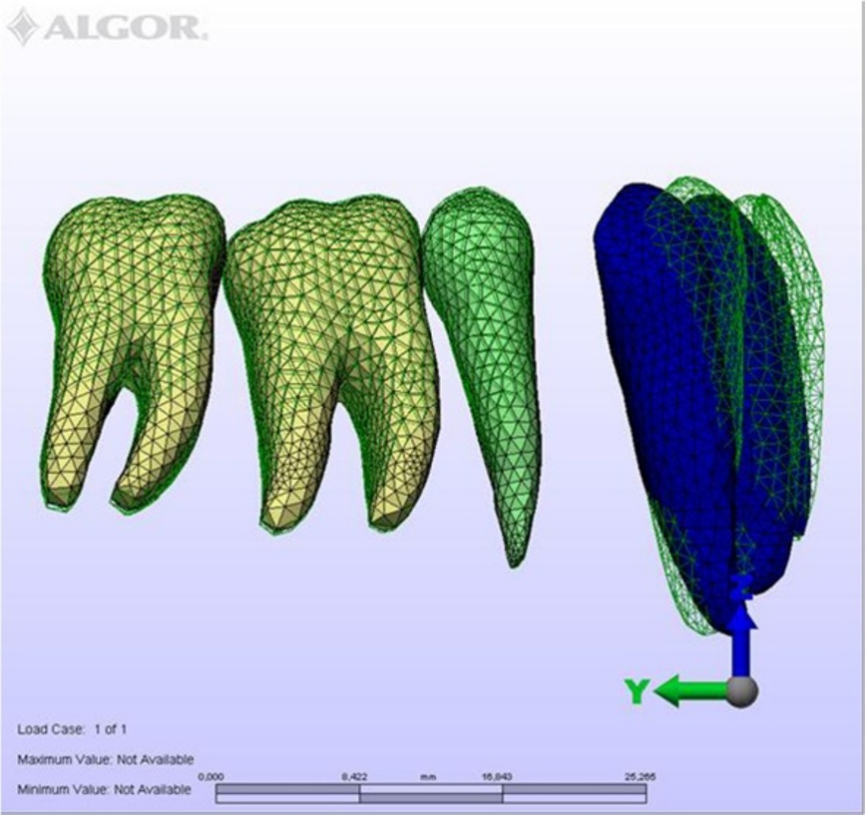
***En masse*** **retraction in the labial technique.**

**Figure 4:**
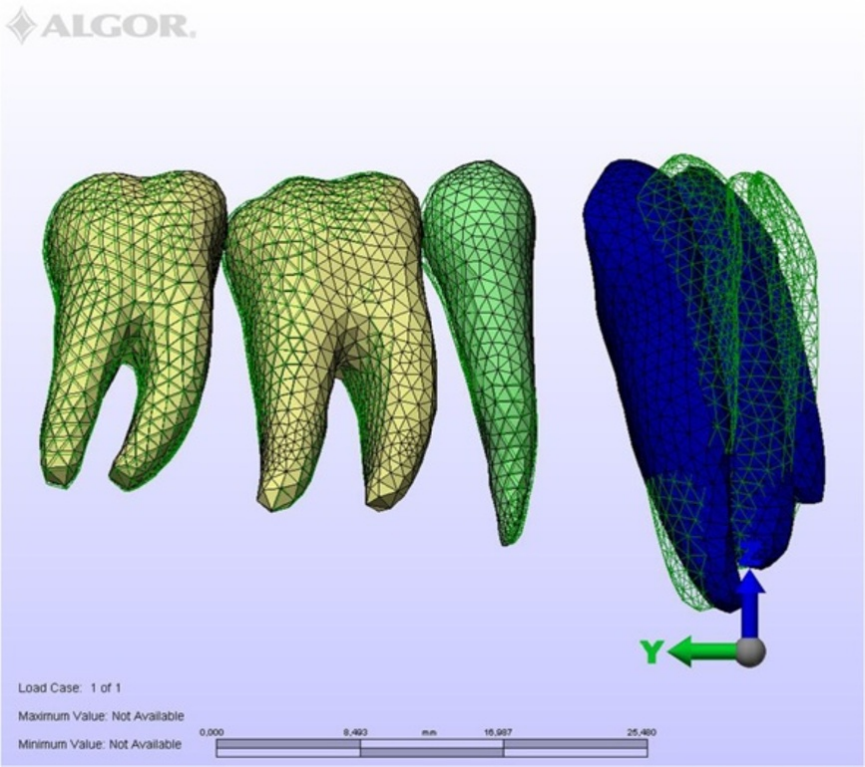
***En masse*** **retraction in the lingual technique.**

Sliding mechanics were used during *en masse* retraction of the anterior dentition, using 0.016 × 0.022-in. SS labial and lingual archwires in the respective slots and applying a 300-g distal force on both sides of the dentition, from the distal wing of canine bracket to the mesial wing of the second premolar bracket in the labial simulation (Figure 3) and between the hooks on the canine and second premolar brackets in the lingual simulation (Figure 4).

A coordinate system with *X*, *Y*, and *Z* axes perpendicular to one another was used, the *X* axis to represent the bucco-lingual direction (+lingual, −buccal), the *Y* axis the mesio-distal direction (+mesial, −distal), and the *Z* axis the vertical direction (+apical, −occlusal). The computer program used to construct the geometric morphology and 3D model was 3ds Max (Autodesk, Inc., Mill Valley, CA, USA). The finite element program Algor (Algor Inc., Pittsburgh, PA, USA) was used to calculate the strains and displacements at each nodal point.

**Figure 5:**
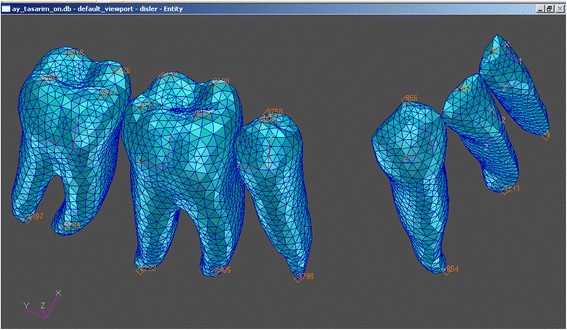
**Location of selected nodes.**

**Figure 6:**
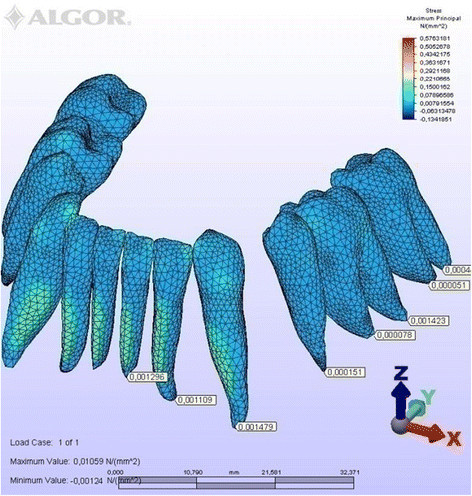
**Maximum principal stresses generated in labial technique (labial view).**

**Figure 7:**
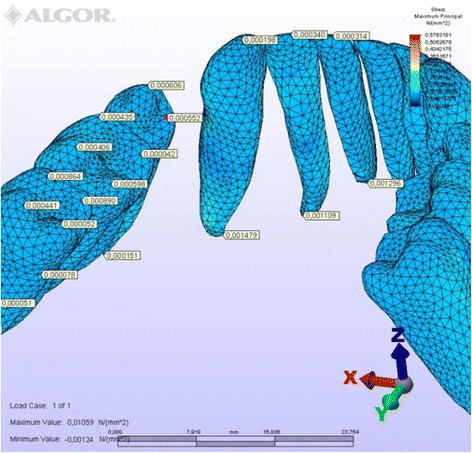
**Maximum principal stresses generated in labial technique (lingual view).**

**Figure 8:**
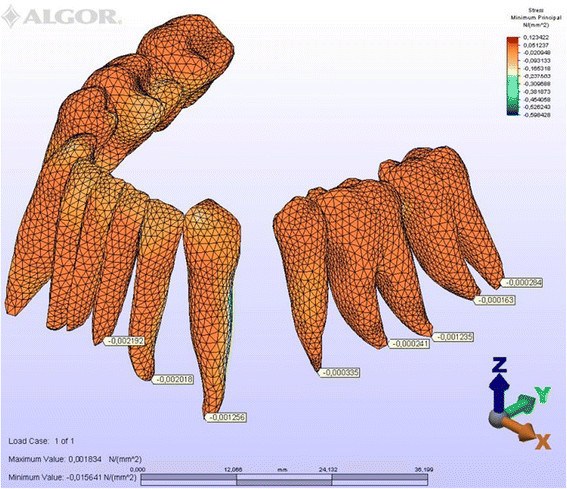
**Minimum principal stresses generated in labial technique (labial view).**

**Figure 9:**
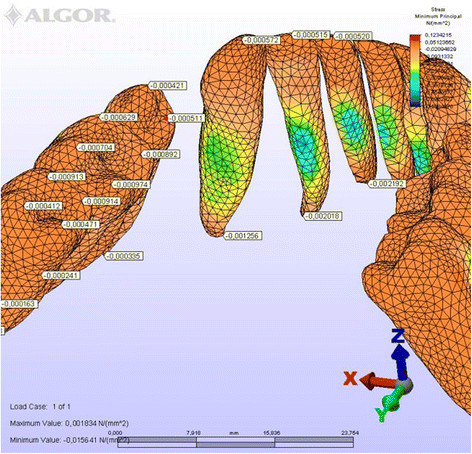
**Minimum principal stresses generated in labial technique (lingual view).**

**Figure 10:**
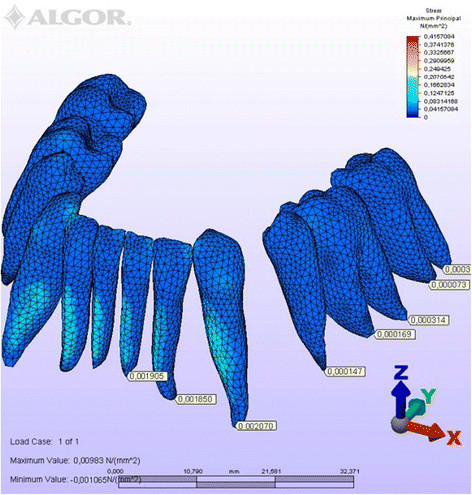
**Maximum principal stresses generated in lingual technique (labial view).**

**Figure 11:**
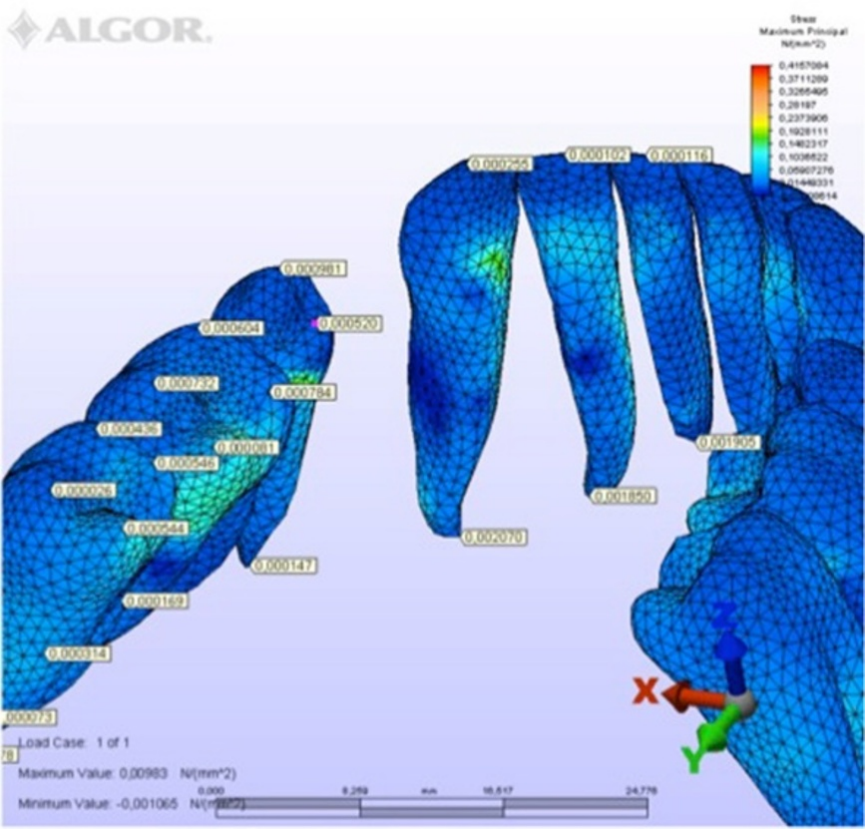
**Maximum principal stresses generated in lingual technique (lingual view).**

**Figure 12:**
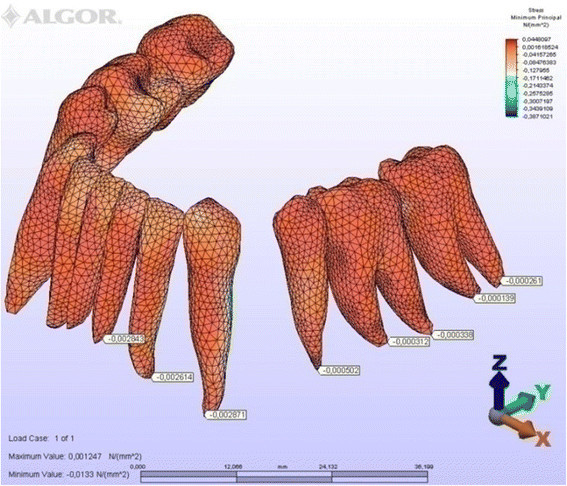
**Minimum principal stresses generated in lingual technique (labial view).**

**Figure 13:**
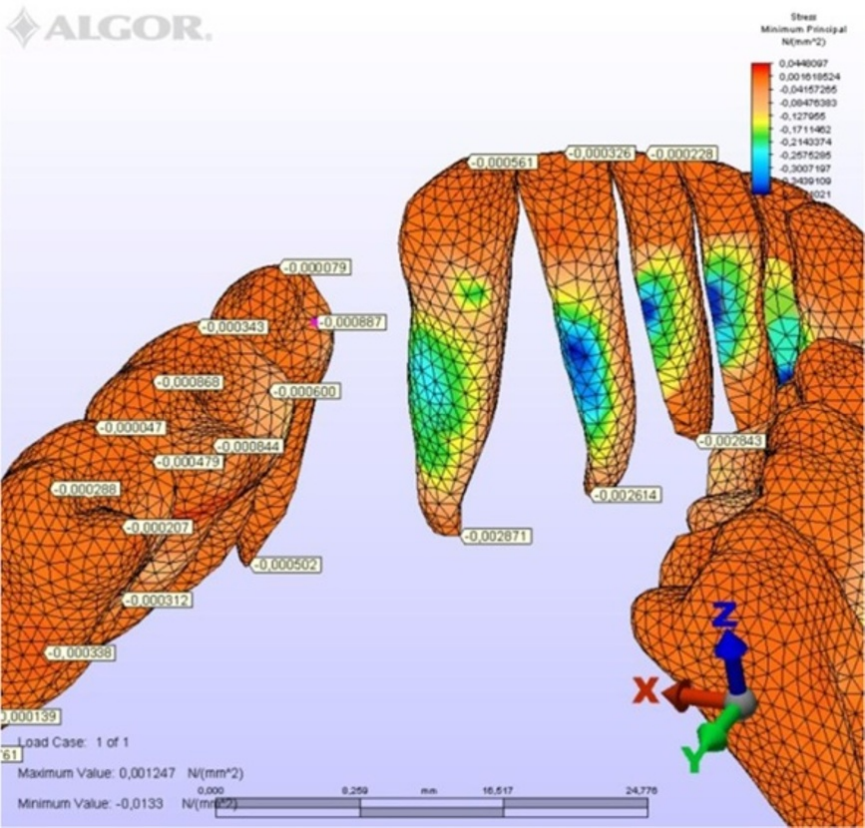
**Minimum principal stresses generated in lingual technique (lingual view).**

To simplify the expression of tooth displacements, reference nodes were placed on the crowns and roots (Figure 5). The amount of initial displacement of these landmark nodes on the *X*, *Y*, and *Z* axes after orthodontic force application was analyzed by FEM, magnifying them by 10,000 for ease of interpretation. The distribution of compressive and tensile stresses occurring at the root surface (Figures 6, 7, 8, 9, 10, 11, 12, 13) was mapped at maximum and minimum principal stresses; the area displaying the maximum positive principal stress was considered the area of maximal tensile stress, and the minimum negative principal stress area was taken as that of the maximum compressive stress.

## Results

The initial displacement of the reference nodes (*X*, *Y*, and *Z* coordinates) caused by the retraction force applied were the following (Table 2):

Central and lateral incisors: lingual (+*X*) and mesial (+*Y*) tipping and extrusion (−*Z*) of the crowns are evident in both groups, although greater tipping and less extrusion occurred with the lingual technique.

Canines: lingual (+*X*) and distal (−*Y*) tipping and intrusion (+*Z*) of the crowns were seen in both groups, although less lingual tipping and more distal tipping and extrusion occurred with the lingual technique.

Second premolars: lingual (+*X*) and mesial (+*Y*) tipping and coronal intrusion (+*Z*) occurred with the labial technique, whereas labial (−*X*) and mesial (+*Y*) tipping plus coronal extrusion (−*Z*) occurred with the lingual technique. Transverse, vertical, and sagittal displacements were smaller, but rotational movement was greater with the lingual technique.

First molars: lingual movement (+*X*) of the crowns with mesio-lingual rotation, mesial tipping (+*Y*), and intrusion (+*Z*) occurred with the labial technique, as compared to labial movement (−*X*) with mesio-labial rotation, mesial tipping (+*Y*), and intrusion (+*Z*) with the lingual technique. Rotational movement was prominent with the lingual technique, while mesial tipping was greater with the labial technique.

Second molars: lingual movement (+*X*) of the crowns with mesio-lingual rotation, mesial tipping (+*Y*), and extrusion (−*Z*) occurred with the labial technique, whereas lingual movement (+*X*) accompanied by mesio-labial rotation, mesial tipping (+*Y*), and extrusion (−*Z*) of the distal cusps was seen with the lingual technique. Once again, rotational movement was more evident with the lingual technique, whereas mesial tipping was greater with the labial technique.

**Table 2:** **Initial displacement of selected nodes on** ***X*** **,** ***Y*** **, and** ***Z*** **axes after application of retraction force (×10** ^**−4**^ **mm)**

		***X***axis		***Y***axis		***Z***axis	
		Labial	Lingual	Labial	Lingual	Labial	Lingual
Central incisor	Occlusal	25.36	48.24	1.28	2.29	−2.86	−2.34
	Apex	−11.28	−22.67	−0.98	−3.66	−2.24	−3.98
Lateral incisor	Occlusal	30.45	51.28	2.5	3.58	−2.53	−1.35
	Apex	−12.34	−23.35	−1.87	−3.45	−1.22	−1.48
Canine	Occlusal	25.59	22.67	−22.05	−25.73	5.53	11.23
	Apex	−11.02	−10.85	8.25	9.97	6.29	9.74
Second premolar	Buccal cusp	18.97	−15.76	19.87	15.66	9.58	7.53
	Lingual cusp	16.24	−12.56	13.43	21.62	11.26	8.25
	Apex	−4.52	−15.73	−10.52	−9.87	6.08	4.42
First molar	Mesio-buccal cusp	12.89	−12.28	9.51	7.98	5.46	4.53
	Disto-buccal cusp	6.78	7.34	10.23	8.25	1.89-	2.38
	Mesio-lingual cusp	10.67	−11.46	5.03	9.34	6.67	4.86
	Disto-lingual cusp	4.54	3.27	5.85	9.67	2.55^-^	3.06
	Mesial apex	−2.76	−7.54	−4.32	−3.87	−1.02	2.54
	Distal apex	−4.66	4.75	−1.23	−3.63	−4.23	−1.18
Second molar	Mesio-buccal cusp	2.43	2.23	7.03	5.87	−2.07	0.57
	Disto-buccal cusp	1.56	4.98	8.25	6.54	−4.65	−3.39
	Mesio-lingual cusp	3.84	3.66	3.84	8.58	−2.46	1.14
	Disto-lingual cusp	2.71	4.85	4.15	8.55	−4.58	−2.02
	Mesial apex	−1.98	3.45	1.84	−2.67	−4.75	−2.28
	Distal apex	−3.25	4.22	2.42	−2.93	−5.03	−2.44

*X*: +lingual, −vestibular; *Y*: +mesial, −distal; *Z*: +apical, −occlusal.

### Principal stresses

The highest minimum negative principal stresses (compression) were observed on the lingual root surfaces of the incisors and distal root surfaces of the canines in both techniques, the highest maximum positive principal stresses (tension) being observed on the labial root surfaces of the incisors and on the mesial root surfaces of the canines. At the incisors, the minimum negative principal stresses (compression) were high in both archwires, but were greater in the lingual appliance. At the canines, however, compression stress was higher in the lingual archwire, while the labial archwire displayed greater maximum positive principal stresses (tension). At these teeth, the stresses induced by the lingual technique were greater. In both techniques, the minimum negative principal stresses (compression) generated at the second premolars were high, but once again greater in the lingual technique. As for the first molars, in the labial technique, compression was greater on the mesial root surfaces, while tension was more evident on the distal root surfaces, whereas the virtual lingual appliance produced greater compression on both root surfaces. Finally, both techniques generated greater minimum negative principal stresses (compression) on the mesial surface of the second molar roots and greater maximum principal stresses on their distal surfaces. Induced stresses on the second molars were greater with the labial technique (Table 3).

**Table 3:** **Maximum and minimum principle stress values of the reference nodes from tooth apex (×10** ^**−4**^ **N/mm** **)**

	Maximum principle stress		Minimum principle stress	
	Labial	Lingual	Labial	Lingual
Central incisor	12.96	19.05	−21.92	−28.43
Lateral incisor	11.09	18.5	−20.12	−26.14
Canine	14.79	20.7	−12.56	−28.71
Second premolar	1.51	1.47	−3.35	−5.02
First molar mesial	0.78	1.69	−2.41	−3.12
First molar distal	14.23	3.14	−12.35	−3.38
Second molar mesial	0.51	0.73	−1.63	−1.39
Second molar distal	4.41	3.78	−2.84	−2.61

## Discussion

In premolar extraction treatment, the orthodontist has several options for space closure, but in the lingual technique, *en masse* retraction of the six anterior teeth is preferred. This is because in full canine retraction, the resulting inter-bracket span between the canine and premolar would be very short, and the inset bend of the archwire distal to the canine would interfere with space closure. Furthermore, in terms of aesthetics, adult patients do not like the space produced between lateral incisor and canine after full retraction [13, 14].

In *en masse* retraction, various archwires, such as 0.016 × 0.016- and 0.016 × 0.022-in. SS or TMA, can be used, but we chose to test a 0.016 × 0.022-in. SS archwire to establish a condition similar to that of the labial technique. There are also two types of mechanics for *en masse* retraction: loop mechanics and sliding mechanics. In the first method, the anterior teeth are retracted directly with a T-loop space closing spring, whereas in the second, the anterior teeth are moved together with an archwire guided by the posterior brackets and tubes, by which bodily movement can be easily achieved [15, 16], our reason for testing this type of mechanics.

In general, under *en masse* retraction forces, bodily displacement and tipping, together with vertical and transversal changes, are considered to occur throughout the dentition. However, as orthodontic tooth movement depends on the location of the line of force relative to the center of resistance, the areas of force application will necessarily be different in lingual and labial orthodontics. In this study, under *en masse* retraction forces, adverse transversal and vertical bowing effects were seen in the entire dentition - lingual tipping and extrusion of the anterior dentition occurred with both archwires, although more incisor tipping was evident with the lingual setup. This confirms the greater lingual crown movement of the maxillary incisors previously noted with lingual orthodontics, as compared to labial orthodontics, when identical loads were applied [12]. These findings suggest that the loss of torque control during retraction in extraction patients is more likely to occur in lingual orthodontic treatment.

We also observed less lingual movement of the canines with the lingual archwire than with the labial appliance. This may be due to the transverse bowing effect of the lingual retractional forces, which deliver a certain degree of expansion on the lateral side of the archwire. Regarding the premolars and first molars, intrusive and lingual movements in the labial archwire and intrusive and labial movements in the lingual archwire can be considered as transversal bowing effects. Sung et al. [8] noticed that during canine retraction with the lingual technique, vertical bowing can result from lingual tipping of the incisors and mesial tipping of the molars; transverse bowing can also occur from rotation of the canine and buccal displacement of the premolars. However, Gorman and Richard [17] found statistically significant differences in treatment results between labial and lingual appliances in their analysis of cephalometric measurements.

The premolars and molars were also tipped mesially and rotated buccally in the lingual archwire and lingually in the labial archwire. In other words, with lingual mechanics, movements were more rotational and less mesial, while with the labial technique, movements were less rotational and more mesial. During anterior movement of the second premolars and molars, the greater mesial movement of the distal cusps, as compared to the mesial cusps, noted with the lingual archwire can be attributed to greater amount of clockwise rotation of the crowns under the retraction forces applied to the lingual side.

Scuzzo and Takemoto [2, 18] have previously stated that lingual application of lingual crown torque to the anterior dentition can generate a distal uprighting effect on the posterior dentition, resulting in greater anchorage control. Indeed, in this study, the stress exerted by the lingual bracket system was always greater than that generated by the labial appliance, except at the molars. This was undoubtedly due to the smaller inter-bracket distance in the anterior sector, which results in a greater load on the teeth even if an undersized archwire is used. Vice versa, the interbracket distance at the posterior teeth is the same on both lingual and buccal sides, and therefore the load is lower even if an undersized archwire is used [19].

That being said, lingual appliances generally provide good anchorage control, and most malocclusions can be successfully treated using traditional orthodontic anchorage and by following basic mechanical principles. However, in certain cases, it may be necessary to consider reinforcing anchorage with temporary screw implants [13, 20, 21]. We show that the lingual location of the brackets influences the pattern of tooth movement. Specifically, in order to avoid bowing effects and rotation of the posterior teeth during *en masse* retraction, it is necessary to reduce the retraction force in lingual orthodontics. At the same time, more lingual root torque should be added to the wire by means of vertical and horizontal compensation curves.

Finally, although this study does not provide comprehensive clinical information, considering only initial rather continuous displacement, it does highlight the usefulness and precision of the 3D FEM technique in mapping structural stress in orthodontic simulations.

## Conclusion

Lingual and labial mechanics provoke very different stress patterns and consequently tooth movements. Specifically, considering a first premolar extraction case treated by lingual orthodontics, more tipping and less extrusion occurred at the lower incisors and less lingual tipping and more distal tipping and extrusion at the canines. Furthermore, at the second premolars, transverse, vertical, and sagittal displacements were less pronounced and rotational movement was greater. At the lower first molar, rotational movement was more prominent with the lingual technique, while mesial tipping was greater with the labial technique, whereas at the second premolar, rotational movement was greater with lingual mechanics, while labial mechanics produced greater mesial tipping.

## Authors’ original submitted files for images

Below are the links to the authors’ original submitted files for images. Authors’ original file for figure 1 Authors’ original file for figure 2 Authors’ original file for figure 3 Authors’ original file for figure 4 Authors’ original file for figure 5 Authors’ original file for figure 6 Authors’ original file for figure 7 Authors’ original file for figure 8 Authors’ original file for figure 9 Authors’ original file for figure 10 Authors’ original file for figure 11 Authors’ original file for figure 12 Authors’ original file for figure 13
