# Supplementary figures and images for: 3D FEM comparison of lingual and labial orthodontics in en masse retraction
Source: Prog Orthod. 2014 May 30;15:38. doi: 10.1186/s40510-014-0038-9 (PMC4884016; doi:10.1186/s40510-014-0038-9)

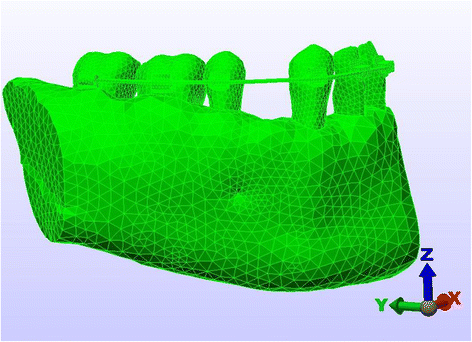

Supplement: Supplementary file 1 — Authors’ original file for figure 1 [file 40510_2014_38_MOESM1_ESM.gif]

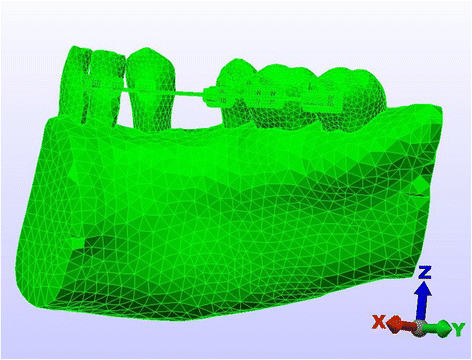

Supplement: Supplementary file 2 — Authors’ original file for figure 2 [file 40510_2014_38_MOESM2_ESM.gif]

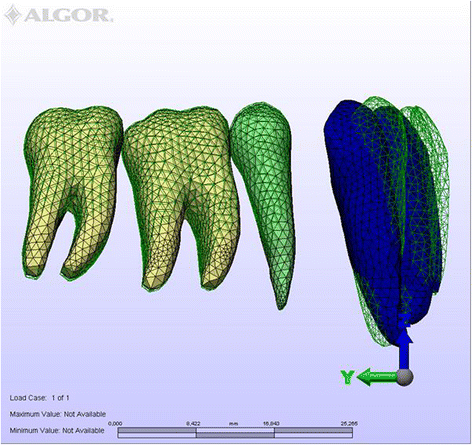

Supplement: Supplementary file 3 — Authors’ original file for figure 3 [file 40510_2014_38_MOESM3_ESM.gif]

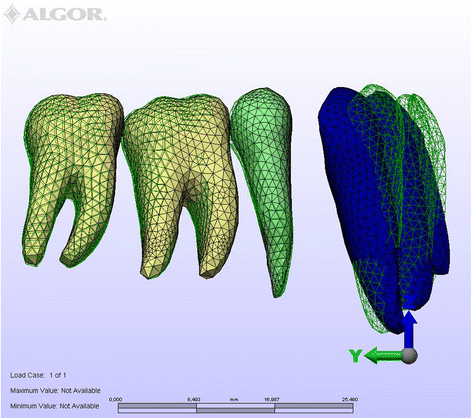

Supplement: Supplementary file 4 — Authors’ original file for figure 4 [file 40510_2014_38_MOESM4_ESM.gif]

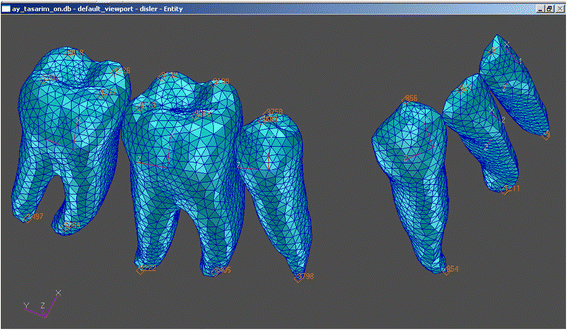

Supplement: Supplementary file 5 — Authors’ original file for figure 5 [file 40510_2014_38_MOESM5_ESM.gif]

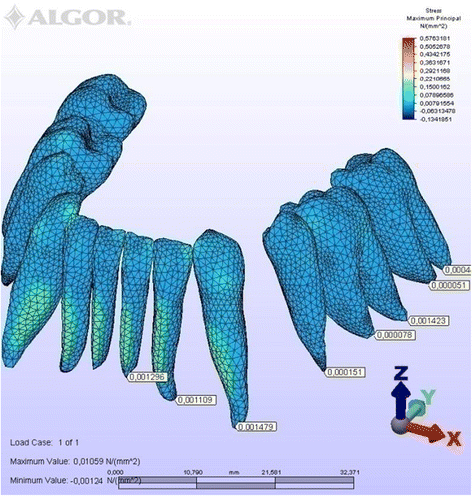

Supplement: Supplementary file 6 — Authors’ original file for figure 6 [file 40510_2014_38_MOESM6_ESM.gif]

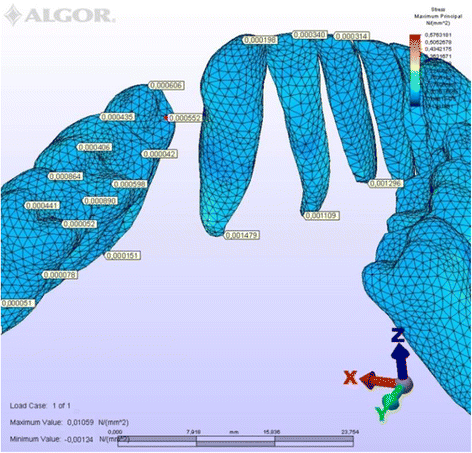

Supplement: Supplementary file 7 — Authors’ original file for figure 7 [file 40510_2014_38_MOESM7_ESM.gif]

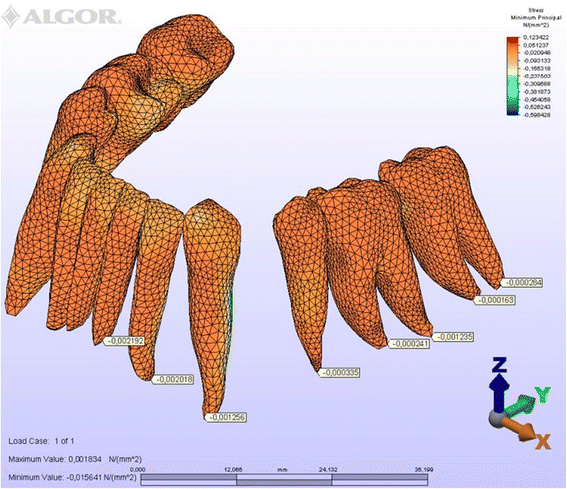

Supplement: Supplementary file 8 — Authors’ original file for figure 8 [file 40510_2014_38_MOESM8_ESM.gif]

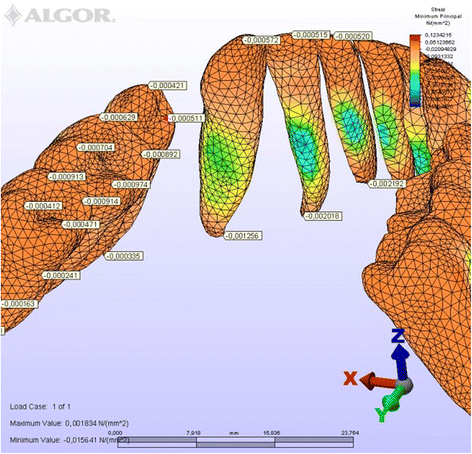

Supplement: Supplementary file 9 — Authors’ original file for figure 9 [file 40510_2014_38_MOESM9_ESM.gif]

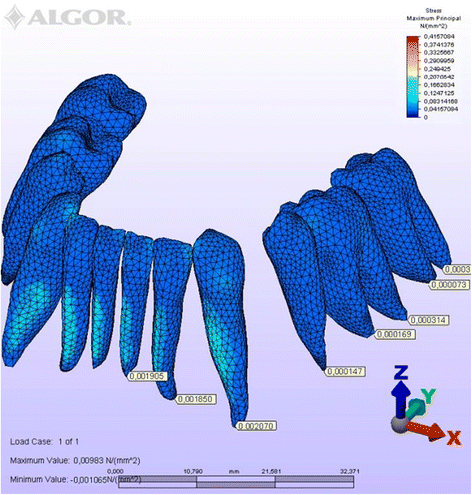

Supplement: Supplementary file 10 — Authors’ original file for figure 10 [file 40510_2014_38_MOESM10_ESM.gif]

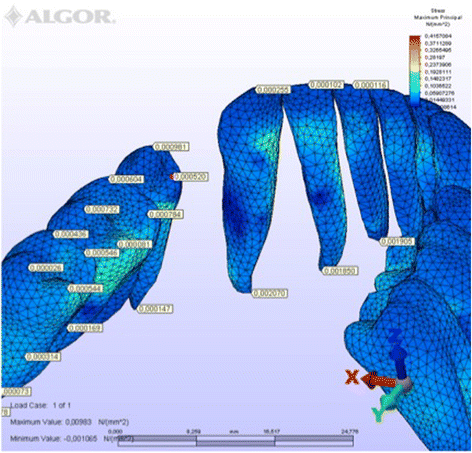

Supplement: Supplementary file 11 — Authors’ original file for figure 11 [file 40510_2014_38_MOESM11_ESM.gif]

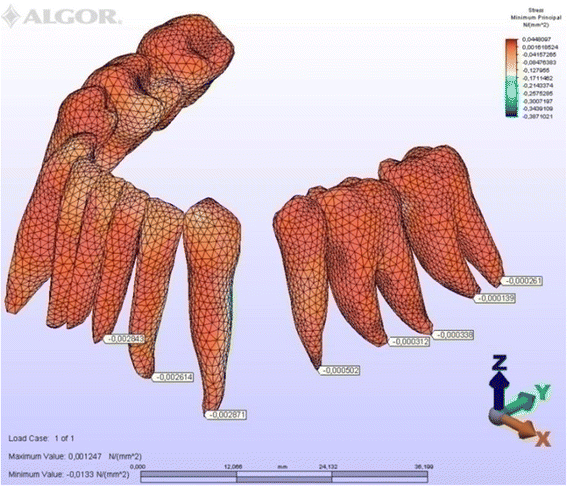

Supplement: Supplementary file 12 — Authors’ original file for figure 12 [file 40510_2014_38_MOESM12_ESM.gif]

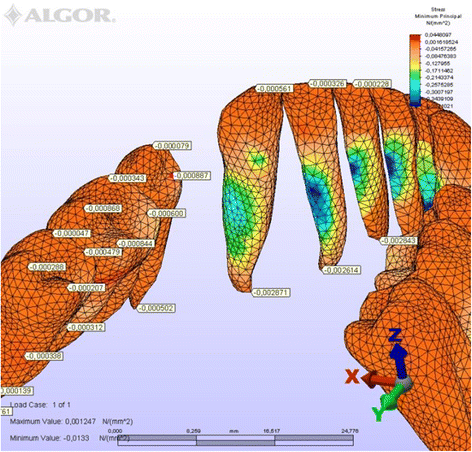

Supplement: Supplementary file 13 — Authors’ original file for figure 13 [file 40510_2014_38_MOESM13_ESM.gif]
